# Access Disparity and Health Inequality of the Elderly: Unmet Needs and Delayed Healthcare

**DOI:** 10.3390/ijerph120201745

**Published:** 2015-02-03

**Authors:** Tetsuji Yamada, Chia-Ching Chen, Chiyoe Murata, Hiroshi Hirai, Toshiyuki Ojima, Katsunori Kondo, Joseph R. Harris

**Affiliations:** 1 Department of Economics, Center for Children and Childhood Studies, Rutgers University, The State University of New Jersey, 311 North 5th Street, Camden, NJ 08102, USA; 2 Department of Epidemiology & Community Health, School of Health Sciences & Practice, New York Medical College, 95 Grasslands Rd., Valhalla, NY 10595, USA; E-Mail: ChiaChing_Chen@nymc.edu; 3 Department of Social Science, Center for Gerontology and Social Science, National Center for Geriatrics and Gerontology, 35 Gengo, Morioka cho, Obu-shi, Aichi-ken, 474-8511 Japan; E-Mail: cmurata@ncgg.go.jp; 4 Department of Civil Environmental Engineering, Iwate University, 4-3-5, Ueda, Morioka-shi, Iwate-ken, 020-8551 Japan; E-Mail: hirai181kan@gmail.com; 5 Department of Community Health and Preventive Medicine, Hamamatsu University School of Medicine, 1-20-1 Handayama Higashiku, Hamamatsu-shi, Shizuoka-ken, 431-3192 Japan; E-Mail: ojima@hama-med.ac.jp; 6 Center for Preventive Medical Sciences, Chiba University, 1-8-1 Inohana, Chuou-ku, Chiba-shi, Chiba-ken, 260-8670 Japan; E-Mail: kkondo@chiba-u.jp; 7 Department of Public Policy and Administration, Rutgers University, The State University of New Jersey, 311 North 5th Street, Camden, NJ 08102, USA; E-Mail: hajoseph@scarletmail.rutgers.edu

**Keywords:** unmet healthcare needs, delayed healthcare, access and health disparity

## Abstract

The purpose of this study is to investigate healthcare access disparity that will cause delayed and unmet healthcare needs for the elderly, and to examine health inequality and healthcare cost burden for the elderly. To produce clear policy applications, this study adapts a modified PRECEDE-PROCEED model for framing theoretical and experimental approaches. Data were collected from a large collection of the Community Tracking Study Household Survey 2003–2004 of the USA. Reliability and construct validity are examined for internal consistency and estimation of disparity and inequality are analyzed by using probit/ols regressions. The results show that predisposing factors (e.g., attitude, beliefs, and perception by socio-demographic differences) are negatively associated with delayed healthcare. A 10% increase in enabling factors (e.g., availability of health insurance coverage, and usual sources of healthcare providers) are significantly associated with a 1% increase in healthcare financing factors. In addition, information through a socio-economic network and support system has a 5% impact on an access disparity. Income, health status, and health inequality are exogenously determined. Designing and implementing easy healthcare accessibility (healthcare system) and healthcare financing methods, and developing a socio-economic support network (including public health information) are essential in reducing delayed healthcare and health inequality.

## 1. Introduction

The number of uninsured population in the United States with access to healthcare services at reasonable costs has been a social and economic problem for their access to healthcare services with reasonable cost. Insufficient insurance coverage, rising healthcare cost and widening disparity in access to healthcare services have adversely affected the elderly population [1,2,3,4]. Today, healthcare costs are generating major problems for welfare states in the short-term and long-term. A major concern is the affordability of healthcare services. A growing number of Americans are delaying or forgoing care due to the financial burden associated with healthcare costs. This concern is compounded by rising healthcare costs, which have led to an increase in medical bill payments from about 15% in 2003 to 19.4% in 2007 [2,5]. Increasing healthcare costs, for which payments include a significant share of out-of-pocket expenses, tend to result in people deferring needed healthcare treatment. The average annual healthcare cost has only been rising: 14.1% in 1999, 15.2% in 2001, to 20% in 2007. In recent years, the average annual rate has slowed relative to the late 1990s and 2000s, but is still expected to grow faster than national income. It is projected healthcare costs will continue to rise 8 percent to 9 percent per year for the foreseeable future.

Disparity in access to healthcare services, under the mixed private and public US healthcare programs, elevates inequality of health and health status among Americans [1,6]. Healthcare disparity is a concern for everyone since it involves a large spread of demographics and a large share in the GDP of the US [7]. Important questions to consider are why have delayed healthcare and unmet healthcare needs been rising; and if financing healthcare is a major issue, what are the factors that can mitigate the burden. A key concern is how healthcare financial burden will be distributed among the different socio-economic status. Finally, this study will explore how healthcare access disparity and health inequality are generated.

Various research literature supports that access disparities lead to unmet healthcare needs [8,9,10,11]. The healthcare disparity gap is a problem that the population will have to disentangle over the next decade. Even though there has been an increase in the managed care system, which has significantly reduced the rate of increased healthcare costs; and there has been an improvement in patient-provider relations, which is related to unmet needs; there is still concern about the disparity in access to healthcare services [10,12,13,14]. There is an appealing and distinctive focus on unmet needs [9,10]. Mollborn *et al.* [9] examined individuals’ trust issues towards physicians. These individuals were ages eighteen years and older, and who had regularly available healthcare providers. Cunningham and Hadley [10] differentiated “delayed care” and “unmet needs” and found individuals’ trust was associated with improved probability of receiving met need care from healthcare providers. Both studies showed uninsured individuals compared to privately insured individuals are more likely to have unmet medical needs. Neither study showed a clear-cut difference regarding healthcare service accessibility, health disparity, and financial burden of healthcare among population.

A focus of healthcare financing characteristics with the sample age groups reveals differences in Medicaid, Medicare, private insurance, and those uninsured. As healthcare costs rises, more of the increasing costs are transferred to certain disadvantaged populations, and patients would have to spend a considerable share of their healthcare costs out-of-pocket. Access disparity for unmet needs and delayed care is relatively complex health policy measures. Studies may convincingly engage with defined measures using concepts from a behavioral model. A study would require a comprehensive analysis of the interfaces and interactions in the nexus of financial characteristics, cognitive aspects, disparity, and inequality.

In order to fill the gap of precise health financial characteristics with individual behaviors, this study employs three dimensions of behavioral measures in addition to socio-demographic factors with different cognitive influences. The three behavioral measures are enabling, predisposing, and reinforcing factors, which are used to determine the influence of health insurance and different cognitive characteristics on unmet needs and delayed healthcare for individuals sixty-five years or older with Medicare. There are three main objectives for this study. First is to shed light on the effects of behavioral characteristics on accessibility of healthcare services, *i.e.*, unmet needs and delayed healthcare services after retirement. Delay in healthcare services is related to compliance with illness treatment, and unique preventive healthcare needs to avoid further health complications. A major goal of the behavioral model is to understand the characteristics of an individual’s access to healthcare. Second, research conducted in this study will offer insight into different health insurance coverage regarding financing aspects: income, out-of-pocket medical costs, Medicare and Medicaid. Furthermore, health insurance includes managed care (HMO) and supplementary Medicare insurance. The public and private health insurance implications are essential to improve the health of the population. Third, this study underscores an improvement in patient trust, patient satisfaction, and effective communication with healthcare providers. In addition, it will lead to a healthier population and decrease unmet needs and delayed care. This will improve the efficiency of delivering healthcare. Clearly this notion is what underlies the broad public uneasiness concerning the health of the population, such as the quality of life as indicated by the absence of access disparity, and also restrain in the rise of medical expenditures under the current complex healthcare system in the United States.

## 2. Brief Background

### 2.1. Medicare Benefits

Medicare benefits consist of two parts: hospital insurance plan, Part A and physician insurance plan, Part B. A person with hospital insurance protection (Part A) may have benefits paid on his/her behalf or, in certain cases, paid to him/her for covered healthcare services. Part B adds additional protection by providing a basic hospital insurance plan and covering a substantial part of physician services in both hospital and nonhospital settings that include an emergency room or outpatient clinic, including same-day surgery, ambulance services, and clinical laboratory. Parts A and B are provided automatically to a person aged 65 or over who is entitled to Social Security Retirement Benefits. The Medicare Part A is financed primarily through a mandatory payroll deduction. Part B is financed through premium payments and contributions from the general fund of the U.S. Treasury.

Beginning in 2006, an additional plan, Part D, has provided subsidized access to prescription drugs insurance coverage. Part D is financed primarily through premium payments and contributions from the Treasury general fund. Part D works differently from Medicare Part A and Part B, beginning with the fact that Part D is an optional plan. However, those eligible need to choose among plans offered by health insurance providers or other companies approved by Medicare. Any person who enrolls in either Medicare Part A or Medicare Part B is eligible to enroll in a Medicare Part D part. If qualified individuals do not enroll in a plan when they first become eligible for Medicare, they might be subject to a Medicare-imposed late enrollment penalty.

### 2.2. Supplemental Insurance for the Elderly

There are many types of private health insurance coverage that the elderly can purchase in order to fill the gaps that are not covered by Medicare. These types of private health insurance/coverage include: employee or retiree coverage from employers and Medigap insurance from private insurance companies. The supplemental insurance pays for parts or all of healthcare costs [15]. These types of private health insurance coverage are known as “supplemental insurance”, “Medicare Supplemental” or “Medigap” insurance is a specific type of private insurance that is subject to federal and state laws. Medicare supplemental insurance policies are sold by private insurance companies to Medicare beneficiaries in order to fill the “gaps” in the original Medicare coverage. There are ten standardized policies, labeled Plan A through Plan J that work in conjunction with the original Medicare plan. Each state decides which of the 10 policies can be sold in the state. Since Medicare does not cover all healthcare expenses, Medicare supplemental insurance is only sold as supplemental health insurance for Medicare recipients. Unlike Medicare, Medicare supplement insurance plan is not a federal program. However, health insurance companies that provide Medicare supplemental insurance coverage are strictly regulated by both the federal and state governments.

### 2.3. Disparity in Health and Accessibility

Improving health status is the central objective of policies designed in the United States today, as emphasized in Healthy People 2020. One of the growing debates in health policy and strategy wages over how to eliminate health disparities [16]. Policymakers often draw a distinction between access and utilization of healthcare services. In particular, the use of healthcare services are discussed in terms of the distribution of healthcare services according to needs of individuals, which brings up the issue of horizontal and vertical equity [14,17,18,19].

Healthy People 2020 defines health disparity as the unequal burden in disease, morbidity and mortality rates experienced by socioeconomic status, race, national origin, and language as compared to the dominant group. There are many reasons for health disparities in the United States. These include lack of access to affordable health insurance coverage, barriers to enrollment in public programs, and patient-provider relation. In addition low health literacy, which is associated with poverty, limited education, and lack of affordable health insurance, is another important factor.

Health disparities are affected by at least two important factors: first, the individual’s ability to produce or maintain their health [20,21] and second, the degree of accessibility of healthcare services associated with unmet needs and delayed care. With regard to the second aspect, which is the main focus of this study, unmet needs and delayed care depend on factors determined by an individual’s choices as well as choices that the individual cannot substantially influence [10,22]. Access to healthcare services, such as the relationship between a patient and his or her healthcare provider, is an important factor that affects individual health disparities [23]. Also, access costs in terms of financial and time costs are factors that influence the amount of healthcare services used by the individual [1,24]. Like ease of access, choosing to have private insurance cover costs of healthcare services is a controllable factor by the individual but the choice of the public health system is not. Both types of health insurances influence the degree of health disparity caused by unmet needs and delayed care. The accessibility of healthcare services for an individual is defined as the availability of private and public health insurance [14,25], ability to procure and use knowledge [26], and other socio-economic and demographic characteristics of the individual [11,27]. The differences in these factors widen the disparity in health among children under the current U.S. healthcare system.

The questions posed in this study are: (1) Do public health programs, such as Medicare and Medicaid, play an effective role in reducing unmet needs and delayed care? (2) How important is the difference of income disparity and out-of-pocket costs in determining the choices made in unmet needs and delayed care situations? (3) What are the influences of predisposing factors such as education, patient trust, patient satisfaction, and patient-provider communication on unmet needs and delayed care? In the remainder of this study, we will strive to provide answers and insights to these inquiries. Section 2 presents the methodology: primary data source, and dependent variables regarding measures of unmet needs and delayed care. The empirical framework includes theoretical based explanations for enabling, reinforcing, and predisposing behavioral factors to evaluate unmet needs and delayed care. In Section 3, we present the empirical results, followed by the conclusion in Section 4.

## 3. Method

### 3.1. Primary Data Source

We used data from the 2003–2004 Community Tracking Study (CTS) Household Survey. The data collection started in February 2003 and was completed in February 2004. The Household Survey contains information from approximately 46,587 individuals in 25,419 families. The individuals ages 16 and older, and represents the national civilian, non-institutionalized population who live in the United States. Non-institutionalized population are individuals who are not in a institution (criminal, mental, or other types of facilities) or an active duty military personnel. The sample is clustered in 60 CTS sites: 51 metropolitan areas and nine nonmetropolitan areas, which were randomly selected to form the core CTS and to be representative of the nation as a whole. The survey was administered by telephone, using computer-assisted telephone interviewing technology. Majority of respondents were selected through list-assisted random-digit-dialing. The CTS Household Survey is the last data with a large sample population and was replaced by the Health Tracking Household Survey (HTHS) with the sample size of 17,797 (16,671) individuals in 9407 (9165) families in 2007 (2010). Both 2007 and 2011 HTHSs focuses on only 12 metropolitan areas unlike, the CTS Household Survey 2003–2004: 51 metropolitan areas and 9 nonmetropolitan areas. The CTS Household Survey 2006 was created before the Medicare prescription program started in 2006. This study reveals the relationship between unmet prescription drugs, perceived delay in healthcare, and unmet needed healthcare. In addition, it evaluates the effect of unmet prescription drug on health before the Medicare prescription program.

The Household Survey instruments covered a large variety of topics including: health insurance, use of health services, and satisfaction with care, health status, and certain demographic information. A family informant provided information on insurance coverage, health resource use, usual source of care, and the general health status of all family members. The informant also provided information on family income, employment, earnings, employer-offered insurance plan, and race/ethnicity of all adult family members. Each adult in the family answered questions from a self-response module that covered the issues of unmet needs, patient trust, satisfaction with physician choice, risky behaviors such as smoking, and the last visit to a doctor.

### 3.2. Dependent Variables: Measures of Delayed Healthcare and Unmet Needs

**Figure 1:**
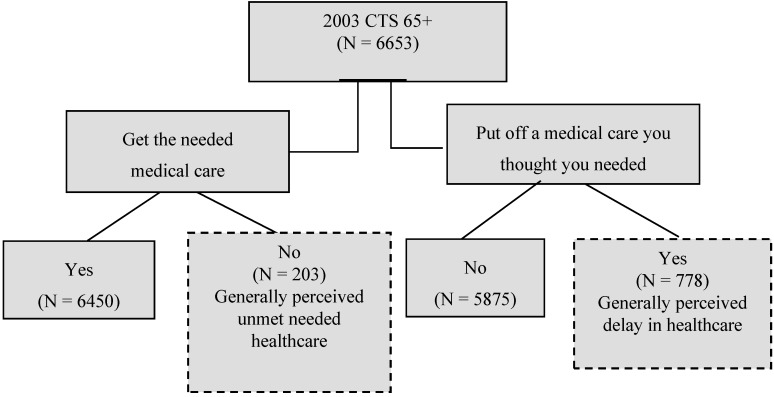
Self-reported generally perceived delay in healthcare and generally perceived unmet needed healthcare: Individuals aged 65 or older in the Community Tracking Survey.

In this study, healthcare accessibility is divided into two dimensions: the general perceived delayed healthcare and unmet healthcare needs. Capturing generally perceived delayed healthcare, the survey included the question, “Was there any time during the past 12 months when you put off or postponed getting medical care you though you needed?” This study then evaluated the different influences of enabling, predisposing, and reinforcing factors on this perceived delay in care. The question about generally perceived unmet needs was “During the past 12 months, was there any time when you didn’t receive the medical care you needed?” However, the information does not provide any information on specific illnesses or conditions. The respondent either recognizes the existence of a health problem or perceives needed care sometime in the near future. The same survey item is used to measure unmet needs [10]. If the response is “yes,” this implies that the person did not get the medical care that he or she needed (see Table 1 and Figure 1). It is measured by patient perceptions of disability, symptom, and diagnoses [28].

**Table 1:** Definitions and Characteristics of Delayed Healthcare, and Unmet Needed Healthcare (aged 65 and over).

Dependent Variables and Definitions	Objectives
*Generally perceived delay in healthcare ^a^*	
Was there ever a time during the past 12 months when you put off or postponed getting medical care you thought you needed?	Evaluate the different influences of enabling, predisposing, and reinforcing on generally perceived delayed care.
*Generally perceived unmet needed healthcare ^a^*	
During the past 12 months, was there ever a time when you did not receive the medical care you needed?	Evaluate the different influences of enabling, predisposing, and reinforcing on generally perceived unmet medical needs.
*Actual unmet needed healthcare ^a^*	
During the past 12 months, did you see a doctor to treat this problem (most recent health problem for which you did not receive or delayed receiving medical care)?	Compare different behavioral differences between generally perceived unmet needs and actual unmet needs.
*Evaluated delayed healthcare (adherence) ^a^*	
Did you see a specialist, get tests, or have a procedure or surgery (If care was delayed, did you put off seeing a specialist or getting tested or getting the procedure or having the surgery)?	Examine the effects of delayed care. Focus more on physician assessments of symptoms and diagnoses.

Notes: The variable of “evaluated delayed care” consists of three types of healthcare services: specialist’s service, tests, and procedures or surgery. ^a^: The symptom response module (SRM) was included in the Community Tracking Study Household Survey, United States. The SRM for specific symptoms are 7 specific symptoms for the serious category that are potentially life threatening if not treated, and 8 specific symptoms for the morbid category that is not life threatening but can potentially have considerable impact on quality of life.

It is crucial to understand that unmet healthcare and delayed care needs are different concepts [9]. In our analysis, four dimensions of healthcare components were evaluated: generally perceived unmet needs, generally perceived delayed healthcare, actual unmet needs, and evaluated delayed healthcare. For generally perceived delayed care, the logical expectation is that self-perceived delayed care at least once in the past twelve months is experienced by putting off the care as displayed in Table 1 and Figure 1. For actual delayed care, a respondent did not receive care after the new occurrence of a specific symptom. Evaluated delayed care is closely related to the kind and amount of treatment that will be provided after a respondent has presented to a medical provider [22,28] as shown in Table 1. The respondent has postponed receiving treatment from a specialist after obtaining a referral from a regular doctor, put off taking a test in spite of the doctor’s recommendation, or deferred having a procedure/surgery in spite of a doctor’s orders. Thus, the evaluated delayed care possibly captures some aspect of adherence. The dimension represents the evaluated side of needs, focusing more on professional assessment of symptoms and diagnoses [28].

### 3.3. Empirical Framework: PP Model

**Figure 2:**
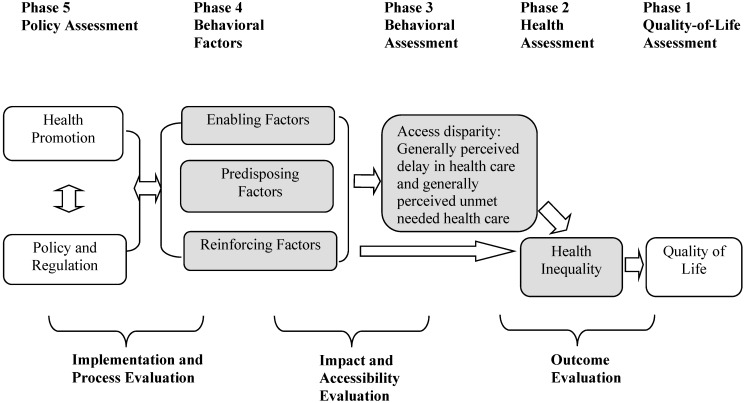
Application of PRECEDE-PROCEED Model to examine generally perceived delay in health care and generally perceived unmet needed health care.

Note: See the adaptation of a model by Green and Kreuter [30].

The line of research on health behavior and its policy implications originated from the PRECEDE-PROCEED model (PP model) [29,30,31]. This offers some concepts and analytical tools to help analyze access disparity and health inequality by using the U.S. Household Survey of the Community Tracking Study. The study applies the PP model to evaluate behavioral characteristics for delayed care, unmet needs, and health inequality between Phases 2 and 4 (Figure 2). Phases 1–5 show the assessment of the PP model in Figure 2. The focus of this study is to assess three categories of influential behavioral factors (enabling, predisposing, and reinforcing factors) by controlling socio-demographic factors in Phase 4 and by evaluating their behavioral influences on delayed care and unmet needs in Phase 3. Phase 5, the policy assessment section, represents the assessment of health promotion, and government policy and regulation on healthcare financing and delivery.

Individual delayed healthcare and unmet needs healthcare can be attributed to the quantity and quality of physical and environmental domains, which can affect health inequality, as well as quality of life. Enabling factors include: access to healthcare services, availability of recourses, and referrals to appropriate healthcare providers, for example income, healthcare financing (out-of-pocket healthcare costs, Medicare HMO, Medicare Supplementary Medical Insurance, and Medicaid), and available methods of healthcare services [30]. Predisposing factors encourage active traits and include: educational level, knowledge, attitude, beliefs, perception, values, culture, *etc.* Attributable to knowledge by higher education, health illiteracy and skills in health information seeking, individuals know how to evaluate quality of health and healthcare [30]. Reinforcing factors are comprised of the different types of feedback, such as the rewards of a particular behavior change made by those in the target population, for example family, marital status, friend, teachers, etc [30].

The PP model offers some concepts and analytical tools that help analyze policy influences on behavioral decisions (see bottom of Figure 2) by using influential factors in Phase 4 within the framework of the PP model. This study assumes that delayed care and unmet needs are measurable as a flow per unit of time because delayed care and unmet needs encompass heterogeneous needs, such as routine, preventative physician care, specialty physician services, acute care, dental care, mental care, and health educational services.

The study assumes that the health inequality in Phase 2 (Figure 2) is attributed to healthcare service use behavior based on his or her delayed care (DELAY) and unmet needs (UNMET) in Phase 3. This is influenced by socio-demographic factors, as well as predisposing, reinforcing, and enabling factors in Phase 4. Individuals with delayed care and unmet needs are evaluated. A functional behavioral model in Phases 3 and 4 could be expressed as follows:

(1) $$DELAY_{i}=\;\xi (EN_{i},\;PR_{i},\;RE_{i},\;SD_{i}\;)\;+\;\omega_{DELAY,\;i}$$

(2) $$UNMET_{i}=\;\text{ϕ}(EN_{i},\;PR_{i},\;RE_{i},\;SD_{i}\;)\;+\;\omega_{UNMET,\;i}$$

Equations (1) and (2) represent the relationship between the health behavioral choice of individual “*i*” and a person with delayed care and unmet needs in Phases 3 in Figure 2. Factors will influence an individual’s delayed care, and ω*DELAY* is an unobserved error, generally assumed to satisfy E(ω*DELAY*| *EN*, *PR*, *RE*, *SD*) = 0. ω*UNMET* is an unobserved error, generally assumed to satisfy E(ω*UNMET*| *EN*, *PR*, *RE*, *SD*) = 0.

Both delayed healthcare (*DELAY*) and unmet healthcare needs (*UNMET*) consist of enabling (*EN*), predisposing (*PR*), reinforcing (*RE*), and socio-demographic factors (*SD*) that will influence healthcare disparity of the elderly. The study incorporates the extended PP Model to observe health inequality and the influential determinants.

In our estimation, enabling factors include income, health insurance related aspects, price of healthcare expenditures (out-of-pocket expenditures), usual source of care, and time related accessibility measures. This study analyzes a variety of health insurance aspects to fill the gap in literature. In order to capture coverage and managed care cost saving aspects, this study includes Medicare HMO, private health insurance with HMO, and Medicare Supplementary Medical Insurance [25,32,33]. In addition to the price of healthcare services, an individual’s income level affects the living standard which contributes to unmet needs and delayed care and, in turn, health outcome [34]. Low income, minority populations received less healthcare than the wealthier population, who has a higher probability of obtaining healthcare when they need it [35]. Various components, as well as the amount of time invested in health services by using usual source of care and time input for accessibility to healthcare services, also influences an individual’s health status through enabling factors as seen in Table 1 [21].

Predisposing factors are education as a substitute for knowledge, patient trust, patient satisfaction, patient-provider communication, and lifestyle risk. The educational level as knowledge is associated with unmet needs and delayed care. Higher education levels affect health capital development and increase efficiency in health status. Years of education represent health capital related to health knowledge, allowing individuals to make better behavioral decisions, which is related to educational attainment [36]. Another barrier to obtaining needed care without delay may be lack of knowledge of the importance of seeing a referred specialist, of getting the recommended test, or receiving proper procedure [37].

There has been a dramatic increase in the interest of measuring patients’ trust in their physicians. Trust is a defining element in any interpersonal relationship and it is particularly central to the patient-provider relationship [38]. Any consideration of the patients’ interpersonal trust in physicians must take into account the general atmosphere in healthcare institutions. Although evidence shows that the majority of patients continue to trust their physicians and health information, concern is growing over the rapid and far-reaching changes in the healthcare system, which have placed great pressure on that trust and may undermine it [39]. The new concerns about patient trust has triggered a recognition that there needs to be a better understanding of the role of trust in a patient-physician relationship and health information, and the relationship between trust and health outcomes.

An increasingly important role in the provision of clinical process and outcomes are related to patient satisfaction through service improvements in nursing homes, hospitals, and clinics. The scope of patient satisfaction includes physician’s inpatient care, primary care services, patient-perceived nurse care, waiting time for services at clinics, *etc.* [40]. Patient satisfaction not only depends on services from healthcare providers but also varies by anxiety level of patient, mental status of patient, and geographic correlation with physician [41,42].

The relationship between patients and their physicians is influenced by lack of cultural competence of physicians to improve communication with patients, lack of knowledge about procedural issues, inconsistencies of recommendation guidelines to patients, lack of physicians’ encouragement of patients’ interest, lack of evidence for benefit from patients’ health insurance coverage, and failure to call back patients for screening. These are all barriers that stem, in part, from the communication between patients and physicians [43,44], and inevitably hinder a patient’s incentive to decrease unmet needs and delayed care. Thus efforts to improve unmet/met needs and delayed care should focus at least in part on improving patient-provider communication. The possible role of health communication between patients and physicians with unmet/met needs and delayed care has recently received greater attention. Health communication is the art and technique of informing, influencing, and motivating individual, institutional, and public audiences about important health issues. Quality oriented communication between patients and their physicians is related to patients’ awareness of unmet needs and delayed care. A lack of communication between a patient and a healthcare provider can lead to serious problems (e.g., chance of survival).

In socio-demographic factors, behavioral aspects of choosing unmet/met needs and delayed care in relation to maintaining good health, use healthcare services which vary by the factors that include age, gender, race/ethnicity, and poverty level. In socio-demographic factors, an individual’s health status based on subjective health is included in a base specification without making a health index because of the lack of ADL objective measurements [45,46]. Estimation is an alternative specification without a health status variable. An underlying factor in the discussion above implies that the estimated coefficient on unmet needs and delayed care would be an upward biased estimate of the true impact of these variables, assuming that individual health status variables are omitted. The results would be biased.

## 4. Results

### 4.1. Reliability of Estimation

For this empirical study, there are three issues of reliability of estimation: exogeneity/endogeneity, multicollinearity, and heteroskedasticity in probit estimation. Health status of the elderly is included in a base specification and educational level, *i.e.*, health knowledge. This is seen as a factor that improves the efficiency with which the elderly can produce better health. The income level of the elderly affects the living standard, which contributes to the health of elderly. In addition, the correlation of educational attainment and income is generally positive. An elderly person with a higher education level is more likely to raise health stock because of his/her health knowledge. Both variables are theoretically important to evaluate the elderly with delayed healthcare services and unmet healthcare needs. Therefore, there are multiple included endogenous variables in this empirical study.

Regarding the issue of exogenity/endogeneity, the study used the Hausman Specification Test to examine the endogeneity of this empirical model, and to examine delayed care, unmet needs, and health variables. Under the null hypothesis, there is no simultaneity and correlation between, health variable and ω*_DELAY_i* which an error term of DELAY Equation (1), and health variable and ω*_UNMET_i* which is an error term of UNMET Equation (2) should be zero, asymptotically. The residual of health is included in the structural form. The study used two instrument variables, body mass index (BMI) and subjective mental health in Table 2. Two of the residuals in the structural equation were not found to be statistically significant at the 10% level, the residual of health (t = −1.42) in the delay of healthcare regression, and the residual of health unmet needs (t = 0.53) in the unmet needs regression. The results imply the health variable for both regressions is exogenous.

**Table 2:** Definitions and characteristics of delayed healthcare, unmet needs needed healthcare, evaluated health status, and others (aged 65+).

Variables	Definition	Mean	Std.	Obs.
**Dependent variable:**				
Generally perceived delay in healthcare ^a^	Was there any time during the past 12 months when you put off or postponed getting medical care you thought you needed? “not get” = 1 and “get” = 0; Min = 0 and Max = 1	0.116	0.321	6668
Generally perceived unmet needed healthcare ^a^	During the past 12 months, was there any time when you didn’t receive the medical care you needed? “yes” = 1 and “no” = 0; Min = 0 and Max = 1	0.030	0.172	6653
Evaluated health status	Composited scales of physical and mental health status. 1 (excellent)–5 (poor), a five-point scale; Min = 2 and Max = 10	4.974	1.697	6591
**Independent variables:**				
Unmet prescription drug	Respondent did not get needed prescription medicines because he/she couldn’t afford it. 1 = couldn’t afford, 0 = otherwise; Min = 0 and Max = 1	0.081	0.272	6663
**Enable factors**				
Income ^b^	Family total income from all sources is equivalized, adjusting for family size by dividing total family income by the squared root of the number of family members (Dollar). Min = 0 and Max = 150,000	33,189	26,702	6853
Poverty level	2002 census family poverty level (Dollar). Min = 8628 and Max = 33,414	11,084.2	3068.2	6853
Out-of-pocket medical costs	Out of pocket medical costs are categorized into six for confidentiality (1–6). Min = 1 and Max = 6	2.696	1.205	6691
Medicare HMO	Medicare coverage at an HMO 1 = coverage, 0 = otherwise; Min = 0 and Max = 1	0.203	0.402	6620
Medicare supplementary medical insurance	An individual with Medicare Supplementary Medical Insurance. 1 = policy coverage, 0 = otherwise; Min = 0 and Max = 1	0.383	0.486	6257
Medicaid	Medicaid coverage. 1 = coverage, 0 = otherwise; Min = 0 and Max = 1	0.0736	0.261	6853
Usual source of care	An individual has a place when he/she becomes sick or needs advice about his/her health. 1 = usual place, 0 = otherwise; Min = 0 and Max = 1	0.934	0.248	6838
**Predisposing factors**				
Education	Number of years of education as knowledge level (years)Min = 6 and Max = 19	12.816	2.967	6853
Patient distrust	3 (very satisfied)–15 (very dissatisfied), composite points. Min = 3 and Max = 15	5.346	2.608	5719
▪ Referral	I think my doctor may not refer me to a specialist when needed. 1(strongly disagree)–5 (strongly agree), a five-point scale; Min = 1 and Max = 5	1.615	1.259	6192
▪ Medical needs	I trust my doctor to put my medical needs above all other consideration when treating my medical problems. 1 (strongly agree)–5 (strongly disagree), a five-point scale; Min = 1 and Max = 5	1.300	0.762	6391
▪ Influence	I think my doctor is strongly influenced by health insurance company rules when making decisions about my medical care. 1 (strongly disagree)–5 (strongly agree), a five-point scale; Min = 1 and Max = 5	2.455	1.615	5398
Patient dissatisfaction	3(very satisfied)–15 (very dissatisfied), composite points Min = 3 and Max = 15	3.923	1.618	3721
▪ Healthcare	Satisfaction with healthcare services. 1(very satisfied)–5 (very dissatisfied), a five-point scale; Min = 1 and Max = 5	1.362	0.781	6577
▪ Primary care doctor	Satisfaction with choice of primary care physician. 1 (very satisfied)–5 (very dissatisfied), a five-point scale; Min = 1 and Max = 5	1.298	0.728	6573
▪ Specialist	Satisfaction with choice of specialist physician. 1 (very satisfied)–5 (very dissatisfied), a five-point scale; Min = 1 and Max = 5	1.273	0.698	3786
Patient-provider miscommunication	3(very satisfied)-14 (very dissatisfied), composite points Min = 3 and Max = 14	4.680	1.799	5961
▪ Listening	How would you rate how well your doctor listened to you? 1(excellent)–5 (poor), a five-point scale; Min = 1 and Max = 5	1.759	0.890	6008
▪ Explaining	How would you rate how well the doctor explained something? 1 (excellent)–5 (poor), a five-point scale; Min = 1 and Max = 5	1.780	0.899	6011
▪ Difficulty	How often did you have a hard time speaking with or understanding a doctor or other health provider? 1 (never)–4 (always), a four-point scale; Min = 1 and Max = 4	1.144	0.499	6041
**Reinforcing factors**				
Marital status	married = 1, otherwise = 0; Min = 0 and Max = 1	0.575	0.494	6853
**Socio-demographic factors**				
Age	Respondent age (years). Min = 65 and Max = 91	74.100	6.618	6853
Gender	Gender: male = 1, female = 0; Min = 0 and Max = 1	0.424	0.494	6853
Race				
▪ White	Race: White = 1, otherwise = 0; Min = 0 and Max = 1	0.840	0.366	6853
▪ African American	Race: African American = 1, otherwise = 0, African American (omitted variable); Min = 0 and Max = 1	0.079	0.270	6853
▪ Hispanic	Race: Hispanic = 1, otherwise = 0; Min = 0 and Max = 1	0.050	0.218	6853
▪ Other	Other races = 1, otherwise = 0; Min = 0 and Max = 1	0.029	0.169	6853
Health status (ill health)				
▪ Physical health for the evaluated health status	Self-reported values of general health status 1 (excellent)–5 (poor), a five-point scale; Min = 1 and Max = 5	2.788	1.125	6853
▪ Mental health for evaluated health status and an instrument variable	Have you felt calm and peaceful during the past 4 weeks? 1 (all the time)–5 (non of the time), a five-point scale; Min = 1 and Max = 5			
▪ Objective health status: BMI, and an instrument variable	Body mass index. Min = 18 and Max = 40	26.709	4.744	6468

Notes: ^a^: The symptom response module (SRM) was included in the Community Tracking Study Household Survey: United States. The SRM for specific symptoms are 7 specific symptoms for the serious category that are potentially life threatening if not treated, and 8 specific symptoms for the morbid category that is not life threatening but potentially have considerable impact on quality of life. ^b^: See Murata *et al.* [46].

The hypothesis that both the coefficient on the health status and the coefficient on education are zero is an example of a joint hypothesis on the coefficients in the multiple regressions (1 and 2). The regressors are possibly multicollinear and linear relationship among some or all-explanatory variables of a regression model makes precise estimation difficult. The variance-inflation factor (vif) detects multicollinearity by using condition indices. The variance-inflation factor (vif) for the general perceived delayed healthcare range from 1.02 to 2.28 in Table 3, that of general perceived unmet needed healthcare ranges from 1.02 to 2.28 in Table 4; evaluated subjective health for the elderly of general perceived delayed healthcare ranges from 1.02 to 2.30 in Table 5; and evaluated subjective health for the elderly of general perceived unmet needed healthcare ranges from 1.02 to 2.30 in Table 6. All vifs are less than 10. As a rule of thumb, when analyzing standardized data, a VIF < 10 indicates a non-harmful collinearity [47,48,49].

**Table 3:** Generally perceived delay in healthcare: Results of probit estimation (aged 65+).

Independent Variables	Estimate	Robust Standard Error	Marginal Effect
Unmet prescription drug	0.402 ^a^	0.104	0.089 ^a^
**Enabling factors: healthcare financing**			
Income	−2.85e−06 ^b^	1.37e−06	−5.19e−07 ^b^
Poverty level	1.58e−05	0.12e−05	2.89e−06
Out-of-pocket medical costs	0.048 ^c^	0.025	0.008 ^c^
Medicare HMO	−8.67e−04	0.069	−1.58e−04
Medicare Supplementary Medical Insurance	−0.031	0.066	−0.005
Medicaid	0.018	0.147	0.003
Usual source of care	0.287	0.179	0.044
**Predisposing factors**			
Education	0.004	0.013	0.80e–03
Patient distrust	0.033 ^b^	0.014	0.006 ^b^
Patient dissatisfaction	0.110 ^a^	0.020	0.020 ^a^
Patient-provider miscommunication	−0.95e−03	0.020	−1.74e−04
**Reinforcing factors**			
Marital status	−0.035	0.073	−0.006
**Socio-demographic factor**			
Evaluated subjective health (ill health)	0.098 ^a^	0.020	0.017 ^a^
Age	−0.010 ^c^	0.005	−0.001 ^c^
Gender (male)	−0.132 ^c^	0.070	−0.023 ^c^
Race			
▪ White	−0.037	0.147	−0.006
▪ Hispanic	−0.128	0.219	−0.021
▪ Others	0.039	0.241	0.007
▪ African American (omitted variable)	--	--	--
Constant	−2.029 ^a^	0.542	---
Number of obs. = 2831			
Log likelihood = −957.931			
Wald Statistic (19) = 161.32			
Probability > chi-square = 0.0000			
Pseudo R^2^ = 0.0853			

Notes: ^a^, ^b^, and ^c^ represent statistically significant levels of probit coefficients as follows: 99% level (^a^), 95% level (^b^), and 90% level (^c^) for a two-tailed test.

All of the results reported in Table 3, Table 4, Table 5 and Table 6 use heteroskedasticity-robust standard errors since increasing variances of distribution for the estimators causes bias and affects efficiency of regression estimation, especially a cross-sectional study with the large data. Therefore, heteroskedasticity does not threaten the internal validity of the multiple regression analysis with the definition of variables in Table 2 by using the process of heteroskedasticity-robust standard errors. Evaluated subjective health status (ill health) will lead to an increase in healthcare service utilization that is also related to a rise in healthcare expenditures. Consequently, this leads to higher healthcare costs, financial problems, delayed healthcare and unmet needs. The elderly with delayed and unmet needs for healthcare may cause health status to weaken. The study of health behavior, by applying the extended PP Model, requires exogeneity/endogeneity tests for delayed care and unmet needs with health variables.

To determine whether the items in each factor could be used for probit regression, dimensionality and reliability (eigenvalue greater than one rule) and consistent estimate of reliability (Cronbach α): the interrelated issues of dimensionality and reliability of responses to a scale, were performed. In Table 2, Table 3, Table 4, Table 5 and Table 6, three items for patient distrust are referral, medical needs, and influence; those of patient dissatisfaction are healthcare, primary care doctor, and specialist; and those of patient-provider miscommunication includes the key items of listening, explaining, and difficulty with communication or understanding. Using the item reduction method (*i.e*., factor analysis), the initial eigenvalues for all three factors were greater than 1, and the eigenvalues dropped toward later components (from 1.453 to 0.864 for patient distrust, from 1.587 to 0.795 for patient dissatisfaction, and from 1.809 to 0.945 patient-provider miscommunication). This implies that there is a uni-dimension of these three factors. To evaluate consistent estimate of reliability of the scale, Cronbach’s coefficient alpha, based on standardized items, was examined for each factor. Cronbach's coefficient alphas are items of patient distrust (α = 0.453 with a poor estimate), items of patient dissatisfaction (α = 0.582 with a questionable level), and items of patient-provider miscommunication (α = 0.667 with an acceptable estimate).

### 4.2. Regression Results: General Perceived Delayed Healthcare and Unmet Needed Healthcare

The results of regression for this study are: general perceived delayed healthcare (Table 3); general perceived unmet needs (Table 4); evaluated subject health of the elderly of the general perceived delayed healthcare (Table 5); and evaluated subject health of the elderly of the unmet needed healthcare (Table 6). The summary statistics and definitions of the variables used in this study are listed in Table 2. The results of these estimations show that the general perceived delayed healthcare and the general perceived unmet needed healthcare are sensitive to unmet prescription drugs in Table 3 and Table 4. The general perceived delayed healthcare and the general perceived unmet needed healthcare are more likely increase as an increase in unmet prescription medicines by older adults aged 65 and over. The results show that an increase in the price of drug and out-of-pocket costs imposes a financial burden on prescription drug use of the elderly.

For enabling factors, as stated earlier, an increase in income is statistically significant and negatively associated general perceived delayed healthcare and general perceived unmet needed healthcare in Table 3 and Table 4, while out-of-pocket medical costs have a higher probability of raising the general perceived delayed healthcare and the general perceived unmet needs. The aforementioned results reveal that the financial burden of receiving healthcare services for those aged 65 and over would lead to the general perceived delayed healthcare and the unmet needed healthcare.

Comparing healthcare financing the public programs, Medicare HMO, Medicare supplementary medical insurance, and Medicaid indicates that there is no clear cut behavioral difference in regarding the general perceived delayed healthcare and the general perceived unmet needed healthcare. Table 4 shows that the Medicaid program is associated with an increased probability of the general perceived unmet needed healthcare. According to the definition and the questionnaire about a term “put off receiving healthcare care services” in the Community Tracking Study Household Survey 2003–2004, the items on the general perceived delayed healthcare are attributable to patient side of reasons, not the motives from the healthcare service provider.

**Table 4:** Generally perceived unmet needed healthcare: Results of probit estimation (aged 65+).

Independent Variables	Estimate	Robust Standard Error	Marginal Effect
Unmet prescription drug	0.369 ^b^	0.146	0.017 ^b^
**Enabling factors: healthcare financing**			
Income	1.42e−06	2.18e−06	4.82e−08
Poverty level	0.14e−04	0.16e−04	4.76e−07
Out-of-pocket medical costs	0.082 ^c^	0.044	0.002 ^c^
Medicare HMO	0.092	0.113	0.003
Medicare Supplementary Medical Insurance	−0.100	0.119	−0.003
Medicaid	0.435 ^b^	0.206	0.023 ^b^
Usual source of care	0.466	0.332	0.010
**Predisposing factors**			
Education	0.034	0.022	0.001
Patient distrust	0.072 ^a^	0.020	0.002 ^a^
Patient dissatisfaction	0.215 ^a^	0.028	0.007 ^a^
Patient-provider miscommunication	−0.039	0.031	−0.001
**Reinforcing factors**			
Marital status	0.075	0.128	0.002
**Socio-demographic factor**			
Evaluated subjective health (ill health)	0.126 ^a^	0.035	0.004 ^a^
Age	−0.019 ^b^	0.008	−0.65e−03 ^b^
Gender (male)	0.086	0.116	0.002
Race			
▪ White	−0.155	0.181	−0.006
▪ Hispanic	−0.679 ^c^	0.385	−0.012 ^c^
▪ Others	−0.018	0.349	−0.62e−03
▪ African American (omitted variable)	--	--	--
Constant	−3.794 ^a^	0.875	---
Number of obs. = 2827			
Log likelihood = −297.346			
Wald Statistic (19) = 177.75			
Probability > chi-square = 0.0000			
Pseudo R^2^ = 0.2544			

Notes: ^a^, ^b^, ^c^ represent statistically significant levels of probit coefficients as follows: 99% level (^a^), 95% level (^b^), and 90% level (^c^) for a two-tailed test.

For predisposing factors in Table 3 and Table 4, we employ four measures to evaluate behavioral choice for those aged 65 and over. Interestingly, both the variables of the general perceived delayed healthcare and unmet needed healthcare are positively associated with patient distrust. The coefficients of patient distrust which consist of referral issues, medical needs, and influence of health insurance firms on physician’s decision making for services are more likely to perceive delayed healthcare and unmet needed healthcare. The results of the distrust variables point out some interesting aspects of the behavioral decisions made by the elderly. In predisposing factors, an increase in patient dissatisfaction with healthcare services, which consist of healthcare in general, a choice of primary care, and choice of specialists, tends to raise generally perceived delayed healthcare and generally perceived unmet needed healthcare. However, the coefficients of patient-provider communication, which consists of a physician’s attitude toward listening efforts, clear explanation about illness situation and treatment, and undivided attention to communicate to the patients, do not show statistical significance and do not present a clear cut association with delayed healthcare and unmet needed healthcare.

### 4.3. Regression Results: Evaluated Subject Health for General Perceived Delayed Healthcare and Unmet Needed Healthcare

The results of Table 5 and Table 6 report evaluated subject health, which is a composited scales of physical and mental health status, of the general perceived delayed healthcare or the general perceived unmet needed healthcare age 65 and over. The coefficient of general perceived delayed healthcare with a positive sign in Table 5 shows that elderly persons, who face general perceived delayed healthcare, reduce their health status by 48.8% within the composited health scale between 2 and 10 as compared to elderly persons who do not have any problems of the general perceived delayed healthcare. The similar interpretation is applied to the coefficient of general perceived unmet needed healthcare in Table 6 and its effect is higher among elderly persons with general perceived unmet needed healthcare by 59.6% than elderly persons without any problems of unmet needed healthcare.

The elderly persons who are not able to afford prescription medicine are strongly associated with evaluated health status in both delayed and unmet needed healthcare. The elderly persons who do not get needed prescription medicines are predicted to raise the health of the general perceived delayed healthcare by 44.2% and general perceived unmet needed healthcare by 49.2% more than the elderly persons who do not have those prescription problems. The large impact of the results imply that prescription drug coverage by Medicare is an important factor that influences the health of the elderly who face the general perceived delayed healthcare or unmet needed healthcare.

For healthcare financing factors in Table 5 and Table 6, a negative sign of income variable indicates that an increase in income of the elderly is associated with an increase in the health status of the elderly among the elderly who face general perceived healthcare or unmet needed healthcare (see the definition of factors in Table 2). A positive sign of poverty coefficients shows that an increase in poverty among the elderly of general perceived delayed healthcare or unmet needed healthcare is associated with reduction of their evaluated subjected health status. A rise in out-of-pocket medical costs within the range between 1 and 6 is also associated with the deterioration of evaluated subject health of the elderly who face general perceived delayed healthcare or unmet needed healthcare by an 18.2% and an 18.4% respectively.

Regarding health insurance factors, coefficients of Medicare HMO indicate that the elderly under Medicare HMO are associated with a 12.1% reduction of bad evaluated subjective health of the general perceived delayed healthcare within the range between 2 and 10. Similarly, the Medicare HMO influence on the evaluated subjective health of the elderly of the general perceived unmet needed healthcare is by 13.2%. Compared to fee-for-service of the traditional health insurance, the provision of preventive characteristics by the HMO tends to raise better subjective health status of the elderly with general perceived delayed healthcare and unmet needed healthcare. Furthermore, the positive signs indicate that the elderly with the Medicaid insurance are more likely to have weak adherence to have good evaluated subjective health.

**Table 5:** Evaluated subjective health (ill health): Elderly of generally perceived delay in healthcare (aged 65+)—Results of OLS estimation.

Independent Variables	Estimate	Robust Standard Error
Generally perceived delay in healthcare	0.488 ^a^	0.100
Unmet prescription drug	0.442 ^a^	0.130
**Enabling Factors: healthcare financing**		
Income	−3.93e−06 ^a^	1.26e−06
Poverty level	0.37e−04 ^a^	0.12e−04
Out-of-pocket medical costs	0.182 ^a^	0.024
Medicare HMO	−0.121 ^c^	0.072
Medicare Supplementary Medical Insurance	−0.001	0.061
Medicaid	0.671 ^a^	0.156
Usual source of care	−0.067	0.172
**Predisposing Factors**		
Education	−0.072 ^a^	0.012
Patient distrust	0.026 ^c^	0.014
Patient dissatisfaction	0.080 ^a^	0.025
Patient-provider miscommunication	0.151 ^a^	0.019
**Reinforcing factors**		
Marital status	−0.154 ^b^	0.072
**Socio-Demographic Factor**		
Age	0.016 ^a^	0.005
Gender (male)	−0.227 ^a^	0.064
Race		
▪ White	−0.149	0.139
▪ Hispanic	0.135	0.230
▪ Others	−0.080	0.236
▪ African American (omitted variable)	--	--
Objective health status		
▪ BMI	0.012 ^c^	0.006
Constant	2.888 ^a^	0.567
Number of obs. = 2786		
F statistics (20, 2765) = 28.15		
Probability > F = 0.0000		
R-squared = 0.1740		
Root MSE = 1.5339		

Notes: ^a^, ^b^, ^c^ represent statistically significant levels of OLS coefficients as follows: 99% level (^a^), 95% level (^b^), and 90% level (^c^) for a two-tailed test.

**Table 6:** Evaluated subjective health (ill health): Elderly of generally perceived unmet needed healthcare (aged 65+)—Results of OLS estimation.

Independent Variables	Estimate	Robust Standard Error
Generally perceived unmet needed healthcare	0.596 ^a^	0.214
Unmet prescription drug	0.492 ^a^	0.129
**Enabling factors: healthcare financing**		
Income	−4.21e−06 ^a^	1.27e−06
Poverty level	0.38e−04 ^a^	0.13e−04
Out-of-pocket medical costs	0.184 ^a^	0.024
Medicare HMO	−0.132 ^c^	0.071
Medicare Supplementary Medical Insurance	−0.003	0.061
Medicaid	0.661 ^a^	0.158
Usual source of care	−0.053	0.172
**Predisposing factors**		
Education	−0.072 ^a^	0.012
Patient distrust	0.028 ^c^	0.014
Patient dissatisfaction	0.074 ^a^	0.025
Patient-provider miscommunication	0.155 ^a^	0.019
**Reinforcing factors**		
Marital status	−0.154 ^b^	0.072
**Socio-demographic factor**		
Age	0.016 ^a^	0.005
Gender (male)	−0.241 ^a^	0.064
Race		
▪ White	−0.136	0.140
▪ Hispanic	0.151	0.231
▪ Others	−0.067	0.235
▪ African American (omitted variable)	--	--
Objective health status		
▪ BMI	0.013 ^b^	0.006
Constant	2.860 ^a^	0.567
Number of obs. = 2782		
F statistics (20, 2761) = 27.43		
Probability > F = 0.0000		
R-squared = 0.1659		
Root MSE = 1.5373		

Notes: ^a^, ^b^, ^c^ represent statistically significant levels of OLS coefficients as follows: 99% level (^a^), 95% level (^b^), and 90% level (^c^) for a two-tailed test.

For the influences of predisposing factors such as education, patient distrust, patient dissatisfaction, and patient-provider miscommunication on the subjective health of the elderly of general perceived delayed healthcare or unmet needed healthcare, both results in Table 5 and Table 6 show quantitatively comparable effects and consistent signs. A negative sign of education indicates a strong negative influence of a rise in educational level on an increase in better subjective health. All patient distrust, patient dissatisfaction, and patient-provider miscommunication are positive and raise both general perceived delayed healthcare and unmet needed healthcare. The delayed and unmet needed care deteriorate health of the elderly.

### 4.4. Concentration Curve: Access Disparity and Health Inequality

**Figure 3:**
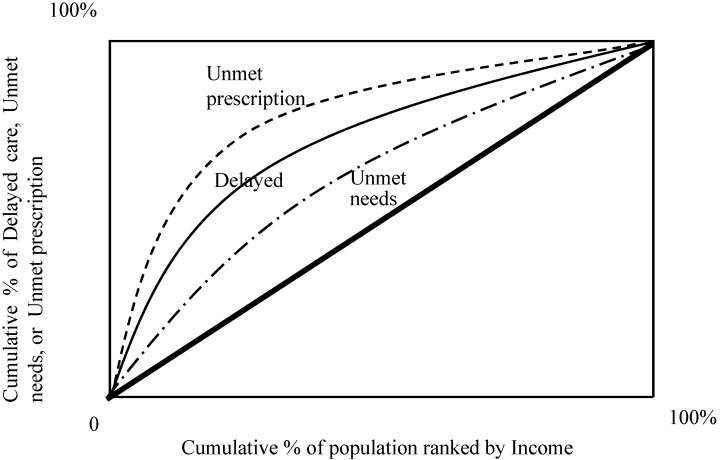
Concentration curves—Access Disparity (Income): Generally perceived delay in healthcare (Delayed care), Generally perceived unmet needed health care (Unmet needs), and Unmet needed prescription drug (Unmet prescription).

Notes: Concentration index: Concentration index using formula/covariance method. Standard error: Standard errors of the concentration index using formula/covariance method. ^a^, ^b^, and ^c^ represent statistically significant levels of 99% level (^a^), 95% level (^b^), and 90% level (^c^) for a two-tailed test.

**Figure 4:**
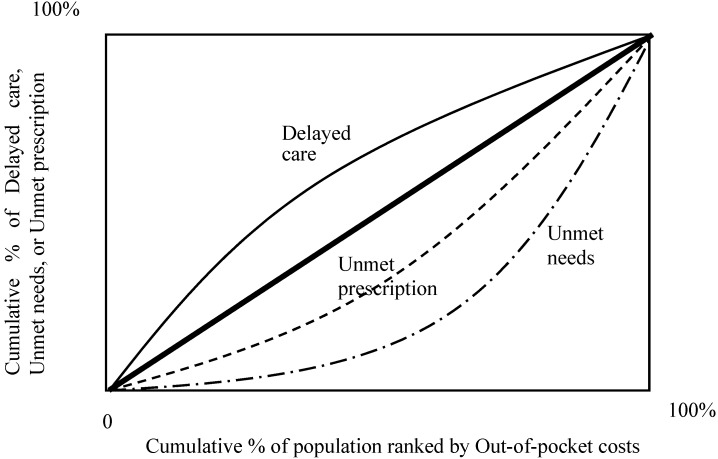
Concentration curves—Access Disparity (Out of Pocket): Generally perceived delay in healthcare (Delayed care), Generally perceived unmet needed health care (Unmet needs), and Unmet needed prescription drug (Unmet prescription) by Out-of-pocket costs.

Notes: Concentration index: Concentration index using formula/covariance method. Standard error: Standard errors of the concentration index using formula/covariance method. ^a^ represents statistically significant levels of 99% for a two-tailed test.

Figure 3 and Figure 4 show the concentration curves which emphasizes and measures health inequalities and identifies inequalities in health by using the concentration index. The index is negative when the curve is above the equality line and positive when the curve is under the equality curve. The concentration index is defined with reference to the concentration curve, which graphs on the x-axis the cumulative percentage of the population ranked by income beginning with the lowest, and on the y-axis the cumulative percentage of “general perceived delayed healthcare,” “general perceived unmet needed healthcare,” or “unmet needed prescription drug,” corresponding to each cumulative percentage of the population of the income in Figure 3. Since all concentration indices take negative values in the table of concentration index if there is access disparity, all concentration curves lies above the line of equality, indicating disproportionate concentration of delayed care, unmet needs, or unmet prescription are among the poor (low income) income population [50,51]. Figure 3 presents a concentration curve for which the curve of unmet prescription is highest among three measures, and unmet prescription is disproportionately large in the population among the poor. The degree to which unmet prescription, delayed care, or unmet needs is more unequally distributed to the disadvantaged of low-income population in the elderly. Access disparity of healthcare services exists among the low-income population.

For Figure 4, the values of “general perceived unmet needed healthcare” and “unmet prescription drug” in the table concentration index for health inequality are positive and their concentration curves lie below the line of equality. However, “delayed care” takes a negative value of the concentration index. The sign indicates that the general perceived delay in healthcare rises as out-of-pocket increases among the elderly. The unmet needed prescription drug is closer to the equality line than the general perceived unmet healthcare needs and the degree of index of the general perceived unmet needed healthcare is smaller than that of the general perceived unmet needed healthcare. The general perceived unmet needed healthcare is disproportionately large in the population among the high out-of-pocket costs.

**Table:** **Concentration Index:** **Access Disparity (Income)**

Accessibility	Concentration index	Standard errors
Delayed care	−0.279 ^a^	0.018
Unmet needs	−0.070 ^c^	0.040
Unmet prescription	−0.290 ^a^	0.021

## 5. Conclusions and Implications

This empirical study concludes unmet needs of prescription drugs for the elderly population have positive influence on the perceived delay in healthcare and perceived unmet needed healthcare. These factors are disproportionally large in the population among the poor (low income) elderly. These three factors raise evaluated ill health status, ranging between 45%~60% among the elderly population. Other interesting findings are that out-of-pocket medical costs elevate perceived unmet needed healthcare and delayed healthcare, and the high out-of-pocket costs raise disproportionally large perceived unmet needed healthcare for elderly population. Our findings also offer insight into the patient-physician relation mechanism. Patient’s distrust and dissatisfaction increase perceived unmet needed healthcare and delay in healthcare. These two factors and patient-provider miscommunication together increase subjective ill health status by about 26%. However, educational attainment of one additional year reduces subjective ill health status by 7%.

**Table:** **Concentration Index:** **Access Disparity (Out of Pocket)**

Accessibility	Concentration index	Standard errors
Delayed care	−0.128 ^a^	0.020
Unmet needs	0.171 ^a^	0.040
Unmet prescription	0.136 ^a^	0.024

Policy makers need to address a diverse amount of issues to make the healthcare more affordable and accessible to reduce inequality of healthcare burden, which in turn lead to health disparity, under the current system. This research reinforces and extends the findings of a previous study for issues concerning health insurance, race, income and satisfaction [9]; and for health insurance and race related aspects [10]. Unlike the results by Mollborn *et al.*, [9] for educational variable, our study supports a hypothesis of educational attainment with better health status for the population aged 65 and over. This study is different since it focuses on understanding the behavioral choices and characteristics of the elderly population aged 65 years and over. In order to underline a more specific policy implication, the behavioral model (the PRECEDE-PROCEED model) was used to comprehend some cognitive aspects, and provide a detailed construction of unmet needs and delayed healthcare. Most importantly, we included a prescription variable that previous studies did not employ.

Our findings show a dimension of increase costs of prescription drug for the elderly in the general perceived delayed healthcare and the elderly in general perceived unmet needs. The results of our study clearly show some important aspects of referral issue and medical needs in distrust category for general perceived delayed healthcare and the general perceived unmet needed healthcare. Our detailed patient satisfaction variables support the result of a single trust variable [9]. In addition, we employed mode detail dissatisfaction measures; composite points by satisfaction with healthcare services, satisfaction with a choice of primary care, and satisfaction with choice of specialists, and underlined the significance of behavioral aspects that the previous study by using a single satisfaction measure [9]. Our results are congruent with the result by Hispanic and African American behavior of unmet needs and delayed healthcare [10]. It is known that economic/financial aspects are debilitating factors for the elderly population falling in the group of delayed healthcare and unmet needed healthcare as a regular source of care. In other words it implies, income and out-of-pocket medical costs are associated with the two characteristics. The income factor is inversely associated with access disparity and health inequality, whereas, the out-of-pocket medical cost has a positive association, or a direct association, with the access disparity and health inequality. Our study shows that lack of financial source of care is associated with an increase in unequally distributed services to the disadvantaged of population of the elderly. The variable of education does not show a clear-cut importance of healthcare knowledge, which is relevant to Grossman’s argument [21].

What is the practical implication of our findings? The government’s Medicaid health insurance is limited to consumer medical care choice that is adversely associated with health and healthcare equality in accordance with our finding from generally perceived unmet needs. An increase in subjective health status with a variable of Medicare HMO partially explains the preventive care aspect to receive meaningful healthcare services in consistent with subjective health of the elderly of the general perceived delayed healthcare and unmet needed healthcare from our finding. Most importantly, medicine prescriptions are a well-known fact and the elderly take multi-prescription to prevent a decrease in health capital from aging. The data for this study is from the Community Tracking Study Household Survey 2003–2004 that was taken before the Medicare prescription program started in 2006. Although the current Medicare prescription program is not ideal one, we still need to do research about the influence of the current Medicare prescription program on the elderly population. When assessing Medicare insurance coverage disparities in access based on unmet needs and delayed healthcare, there is little evidence the effectiveness of policy and healthcare and health disparity among disadvantaged population.

It is not difficult to explain why two of three measures produced the same results of positive influence of patient distrust and dissatisfaction on health of the elderly of the general perceived delayed healthcare and unmet needed healthcare. A recent rapid increase in medical costs associated with an increase in access disparity causes health disparity among the elderly. Further study is strongly recommended to understand and to comprehend characteristic different behavioral choice about delayed healthcare and unmet needed healthcare for reducing health disparity, especially unequally distributed disadvantaged of income population in the elderly and delayed healthcare and unmet needed healthcare for decreasing disproportionately large in the elderly among the high out-pocket costs.

## Endnote

**1:** The concentration index is computed on individual (micro) data. The concentration index is computed as [50,51]: CI = 2cov(h_i_, R_i_)/μ, where h_i_ is the individual i’s indicator variable (e.g., health status) whose inequality is measured, μ is its mean, and R_i_ is the i_th_ individual’s fractional rank in the socioeconomic distribution (e.g., the person’s rank in the income distribution). “cov” is the covariance. Standard errors of CI are obtained by a covariance/formula method:

var(CI) = (1/n)[(1/n)Σ_i = 1_a_i_^2^ − (1 + CI)^2^], where a = (h_i_/μ)(2R_i_ − 1 − CI) + 2 − q_i-1_ − 1 − q_i_, and q_i_ = (1/μn)Σ_j = 1_(h_j_) is the ordinate of the concentration curve CC_h_(p), and q_0_ = 0.
